# Editorial: Hospital management and healthcare policy: financing, resourcing and accessibility, volume II

**DOI:** 10.3389/fpubh.2025.1699910

**Published:** 2025-10-16

**Authors:** Thomas T. H. Wan, Jay Shen, Hanadi Y. Hamadi

**Affiliations:** ^1^School of Global Health Management and Informatics, University of Central Florida, Orlando, FL, United States; ^2^Center for Health Disparities and Research, School of Public Health, University of Nevada in Las Vegas, Las Vegas, NV, United States; ^3^Department of Health Administration, Brooks College of Health, University of North Florida, Jacksonville, FL, United States

**Keywords:** demand management, risk identification and stratification, informatic integration, predictors of health services use, implementation research

## 1 Introduction

As the editors of “FRONTIERS IN PUBLIC HEALTH,” Research Topic—*Hospital management and healthcare policy: financing, resourcing and accessibility, volume 2*, we are pleased to introduce this Research Topic, which highlights pivotal research across multiple critical categories: Healthcare policy, Hospital Management, Healthcare Financing, Healthcare Information, and Health Insurance. Each of these domains represents a cornerstone in the architecture of effective public health systems worldwide. This Research Topic of studies provides invaluable insights into the challenges and innovations shaping the future of global health. This Research Topic brings together a diverse array of studies and analyses that collectively advance our understanding of strategies that could maximize and optimize healthcare organization performance and better outcomes. Each article offers its unique contributions to the ongoing discourse on how to improve public health systems globally. As we continue to face new challenges and opportunities in public health, the research presented here serves as a vital resource for governmental officials, policymakers, practitioners, and scholars dedicated to fostering healthier communities worldwide. Furthermore, the advancement of analytical tools or predictive analytics has shed light on the prospects for conducting value added evaluation of intervention strategies for global public health systems.

## 2 Synthesis of important findings of the 26 articles included in volume 2

### 2.1 Healthcare policies and reforms

Healthcare policy remains at the forefront of addressing disparities and optimizing care. The synthesis of seven studies across diverse health systems reveals how governance mechanisms fundamentally shape system performance and resource allocation outcomes. Technology integration drives governance performance when combined with supportive organizational structures. Big data implementation in Sichuan Province's ethnic areas produced significant public health governance improvements only when paired with favorable ecological conditions and strengthened public opinion management. Technology alone generated limited impact, however. Effective governance required a synergistic integration of technological capabilities, organizational capacity, and environmental support systems. Payment reform fundamentally alters service delivery models across healthcare systems. Medical insurance payment reforms pushed hospitals away from traditional volume-based care toward value-driven service approaches. Fee-for-service models encouraged volume increases while bundled payments promoted efficiency improvements. Successful payment reforms require precise alignment between financial incentives and quality measures. China's National Comprehensive Medical Reform has demonstrated measurable outcomes across 31 provinces, increasing licensed physicians by 12.6% and reducing per capita inpatient costs by 7.2% in pilot regions. Regional integration requires context-specific implementation strategies rather than uniform national approaches. Urban and rural medical insurance integration across Chinese regions showed varying impact intensities depending on local characteristics. Single-tier payment models produced stronger effects than multi-tier systems. Policy impact followed an inverted U-shaped relationship with economic development levels and healthcare resource availability. Regions with moderate development levels achieved optimal integration outcomes. Pharmaceutical innovation policies create competitive pressures that stimulate research investment. China's acceptance of foreign clinical data increased R&D spending among domestic pharmaceutical firms by creating competitive pressures from imported drugs. Companies with stronger absorptive capacity benefited more from the policy. Non-state-owned firms engaged in new drug research showed greater innovation responses than state-owned enterprises. Traditional medicine integration faces economic dependencies that limit coordination development. Traditional Chinese medicine service levels exceeded economic development in most regions of China. Eastern regions achieved higher coordination than central, western, and northeastern areas. Economic development served as the decisive factor in traditional medicine's integration success. Hospital closure patterns reveal governance challenges at system levels. Six key drivers including financial pressures, demographic shifts, and policy changes created spillover effects that reduced quality at remaining facilities. Provider adaptations included reducing service duration rather than expanding capacity. These responses damaged patient outcomes while failing to address underlying capacity constraints. Policy implementation success requires multilevel approaches that address technological, organizational, and environmental factors simultaneously. Tailored implementation strategies based on local contexts show greater success rates than uniform national approaches. Future governance frameworks must account for institutional capacity, political support, and economic conditions that mediate policy transfer processes.

### 2.2 Hospital management and governance

Health management plays a pivotal role in ensuring the efficiency and effectiveness of healthcare delivery. Several important structural and ecological factors have been identified as drivers and critical factors to help optimize the performance of health delivery systems. For example, the German study on hospitalization of patients with type 2 diabetes reports that avoidable hospitalization can be realized if proper procedures for implementing disease management and utilization management strategies are being developed. An Ethiopian study has documented the intrinsic and extrinsic factors related to health workers are important motivating strategies for enhancing job satisfaction and quality of care. Similarly, a case study approach on nurse staffing has shed light on multidimensional optimization measures for establishing flexible and mobile nurse staffing. This could be an important quality management strategy for achieving optimal staffing and nursing care. In a study of operational efficiency of 16 traditional Chinese medicine hospitals in Zhengzhou, China, a power application of data envelop analysis (DEA) has demonstrated that a half of 16 hospitals were identified as having a lower level of efficiency measures. Thus, mechanisms and directions for improving efficiency are noted. Spatial effects of Chinese township health centers in rural areas are documented in a longitudinal study of health resource allocation. This report also highlights important governmental roles in making equitable resource allocations. Similarly, better quality improvement strategies are needed to reduce child mortality in middle and lower levels of resource countries. In summary, the cited six studies on health management and governance have suggested the path for optimizing the quality of care via improvement of technical and operational efficiency of healthcare organizations.

### 2.3 Healthcare financing, insurance, and cost effectiveness analysis

Health insurance is a fundamental pillar of universal health coverage. The appraisal of universal health insurance and maternal health services utilization before and after the implementation of Jaminan Kesehatan Nasional (JKN) in Indonesia provides critical insights into the impact of health insurance policies on healthcare access and outcomes. Another example of universal coverage is Thailand's Universal Coverage Scheme (UCS). Its experience shows that although 75% of the population are covered by UCS, many healthy young people, who have jobs with high income and reside in urban areas, do not enroll in or use UCS, mainly due to long waiting queues, inconvenient office hours, and quality of care of those healthcare facilities covered by UCS; those people often choose either private-providers or self-medication.

Health insurance and payment reforms have occurred in some countries in recent years, with varied degrees of success. Analyzing the China Household Tracking Survey (CFPS) data reveals that the implementation of the catastrophic insurance program significantly reduces financial burdens of healthcare for both urban and rural residents, with rural residents, high-income groups, and non-older adult populations being benefited more, indicating that the urban-rural gap in financial burdens due to severe illnesses has been narrowed in China. A study examines the 2011–2019 data of 31 European Union countries reports that public finance management focusing on the identification of policy priorities is associated with the reduction of pre-mature mortalities for both men and women although mortality rates in both sexes vary cross different counties that have different mortality rates. Taiwan's experience in implementing global budget under a single-payer system shows associations between hospital beds per population and hospital admission through emergency department (ED) among patients with diabetes-related ambulatory care sensitive conditions (ACSC). The findings indicate that under hospital global budgeting with a floating-point value mechanism, more hospital beds likely motivate hospitals to admit ED patients with ACSCs, suggesting urgent needs to add value-based incentive mechanisms to the existing global budget program to encourage healthcare providers focusing on quality of care rather than volume generating. In fact, a Swedish study has reported positive effects of value-based reimbursement on healthcare cost, showing 11 percent decrease per episode of care (i.e., inpatient and outpatient care) among patients undergoing lumbar spine surgeries, resulting from better coordinated post-discharge care.

Several studies in China have also evaluated cost effectiveness of health services in such areas as disease screening, surgery infection, and cancer treatment. They provide empirical evidence of achieving cost-effectiveness in clinical practice. One study evaluates the point-of-care testing (POCT) glycosylated hemoglobin (HbA1c) screening for Type 2 diabetes in both urban and rural areas of China. Its findings indicate that POCT is more cost-effective than other testing options such as venous blood HbA1c and fasting capillary glucose (FCG), suggesting POCT could be recommended for future clinical practice in China. Another study, based on the 2023 and 2024 data of three tertiary hospitals in Xinjiang, found an average direct economic loss of $1,364 per patient with surgical site infections (SSI) or hospital acquired infections, as compared with their counterparts without any SSI, suggesting a reduction in SSI is likely to improve efficiency of inpatient care. The third study measures the cost-effectiveness of the interventions, quality-adjusted life-year (QALY), and incremental cost-effectiveness ratio (ICER), to compare tislelizumab plus chemotherapy vs. chemotherapy as first-line treatment for extensive-stage small cell lung cancer (ES-SCLC). It, combining the consideration of the wiliness to pay threshold, concludes that tislelizumab plus platinum and etoposide seems to be a cost-effective treatment option for ES-SCLC compared to the standard chemotherapy. The last study uses clinical trials' data to compares the cost-effectiveness of incorporating toripalimab alongside chemotherapy vs. the placebo + chemotherapy option, treating patients diagnosed with metastatic triple-negative breast cancer (mTNBC). The study, with the consideration of the willingness to pay threshold, that toripalimab plus chemotherapy is a more cost-effective strategy than the placebo + chemotherapy option.

## 3 Prospects for developing predictive analytics and innovative healthcare research

The authors of this Research Topic have made substantive contributions to both the theoretical and methodological advancement of healthcare research. Nonetheless, more systematic investigations are needed in health economics and implementation science, particularly through experimental study designs combined with longitudinal analyses of panel data.

The future of global health research depends on building collaborative teams and evaluating healthcare management strategies that improve both efficiency and effectiveness in service delivery.

### 3.1 Approaches

Risk identification and stratification are essential for delivering targeted health services. Demonstration projects should be designed to collect both personal and contextual information, enabling sustainable and cost-effective care models at the population level.

We recommend a two-stage analytic approach to study healthcare utilization and overutilization:

- Stage 1: Automatic interaction detector (AID) analysis
  ° Predictors: demographic, social, and contextual factors (*Xi*).
  ° Outcome: demand for ambulatory care (*Y*).
  ° Objective: to identify mutually exclusive, relatively homogenous subgroups of healthcare users.
- Stage 2: Behavioral predictors
  ° Predictors: motivation for lifestyle change, risk perceptions (*Zj*).
  ° Outcome: use of residual variance of the outcome variable from Stage 1 as the dependent variable.
  ° Objective: to refine prediction of hospitalization or readmission.

This multi-level statistical design can identify at-risk populations, guide innovative interventions, and integrate care for targeted groups. Such an approach yields insight into how individual, societal, and health system factors influence healthcare demand, helping reduce unnecessary utilization, lower costs, and design new capitation models for effective population health management.

Demand drivers. Utilization patterns, particularly under universal health insurance systems, can be tracked through ambulatory and inpatient care. Evidence shows that populations in Asia generally use more services than those in the United States (1), with demand increasing due to aging demographics. This trend threatens sustainability and calls for new cost-containment and care management strategies, including better continuity and coordination of care (1, 2).

Editorial objectives. This editorial seeks to:

1. Identify a population health management approach to control uncontrolled demand growth through risk identification and stratification.
2. Evaluate the effectiveness of care management strategies in addressing challenges such as aging populations, workforce shortages, system inefficiencies, rapid changes or disruptive innovations, and rising home healthcare needs.

Future studies, guided by Andersen's Behavioral Model, should examine:

- Can service use be segmented into homogeneous subgroups to reveal patterns in utilization?
- What behavioral, personal, and social determinants influence service use in each subgroup?
- How do care management strategies affect efficiency and outcomes?
- How can delivery systems be optimized for effectiveness and sustainability?

To answer the above questions, we expand the version of Andersen and Newman's conceptual model of health service utilization to classify the individual, societal, and health system determinants into the predisposing, enabling, and need for care factors (3–7). The analytic methods include: 1) the first stage least squares analysis, using demographic and social factors as predictors of health services to stratify the patient population; 2) the second stage least squares analysis, using behavioral and health status factors as predictors of the residual variance in health services used; and 3) the ability of the difference-in-differences analysis between the pre- and post-intervention periods for multiple subgroups of the targeted population can detect the benefits of multi-care management strategies (8–10).

Health services researchers have consistently documented the need to detect and identify excessive users of health services (11, 12). Interestingly, there is a paucity in the empirical literature revealing standardized criteria to classify the users since there are disparities and variations in health services use at different populations. Table 1 is a summary of relevant predictors or determinants in studying the utilization of physician services and hospitalization. Alkhawaldeh et al. (13) in a systematic review on Andersen's behavioral system model of health services utilization reported that its theoretical focus varies by perspectives and types of healthcare used. However, the consensus among researchers and scholars in health services research suggests that the enabling factors, such as health insurance coverage, income and usual sources of care, exert more influences on the variability in healthcare utilization than the predisposing factors (14). Furthermore, the need for care factors, such as perceived health and clinically determined diagnoses, are more influential than the predisposing and enabling factors (9, 11–13). After twenty years of application of behavioral model, Stein et al. (9) noted that behavioral factors are still relevant predictors of health services use. Table 1 summarizes the actual implementation of varying care management strategies and the impacts on ambulatory care and inpatient care.

**Table 1:** Implementation and evaluation of care management strategies under the National Health Insurance in Taiwan (1995–2025).

**Care management programs and strategies**	**Demand management strategies**	**Utilization management strategies**	**Quality management strategies**	**Engagement management strategies**
Program activities	x	x	x	x
Program proximal outcomes	x	x	x	x
Program intermediate outcomes	x	x	x	x
Program distal outcomes	x	x	x	x

Check mark (x) to implement specifically designed and implemented strategies.

### 3.2 Methodology

#### 3.2.1 Design

Because randomized trials are often impractical in health services research, we propose a quasi-experimental design using pre–post comparisons with control groups that could be matched by using the propensity score matching and analysis. This design allows detection of intervention effects at both patient and provider levels while maintaining ethical feasibility (15, 16).

#### 3.2.2 Measurements

- Dependent variable: healthcare utilization (ambulatory care, home care, nursing home care, hospitalization, etc.).
- Stage 1 predictors: predisposing factors (e.g., age, gender, marital status, rural–urban residence, distance to service centers, types of health insurance system).
- Stage 2 predictors: enabling and need factors, used to explain residual variance from Stage 1.

#### 3.2.3 Analytics

- Stage 1: Automatic interaction detector (AID) or classification and regression tree (CART) methods to identify homogeneous subgroups (e.g., high users).
- Stage 2: Regression models using residual variance from Stage 1 as the dependent variable.
- Difference-in-differences (DID): compares pre- and post-intervention outcomes across groups, controlling for variability with propensity score matching.
- Equation: DID = (b–a)/[(a+b)/2] (b – a)/[(a + b)/2] (b–a)/[(a + b)/2] × 100, where *a* = pre-intervention outcome, *b* = post-intervention outcome.

This quasi-experimental, multi-stage design enables evaluation of the net impact of management interventions, providing actionable evidence for health system innovation.

### 3.3 Implications

Healthcare demand is shaped by eight critical factors:

1. Population aging
2. Workforce shortages
3. Rising chronic disease burden
4. Growth in home healthcare needs
5. Inequities in access to quality care
6. Availability of integrated care
7. Disruptive health information technologies and innovations
8. Develop and use artificial intelligence assisted clinical care support

No single intervention can address these challenges. However, integrated care management strategies—combining demand management, utilization management, quality improvement, and patient engagement—offer a viable path forward.

**Figure 1:**
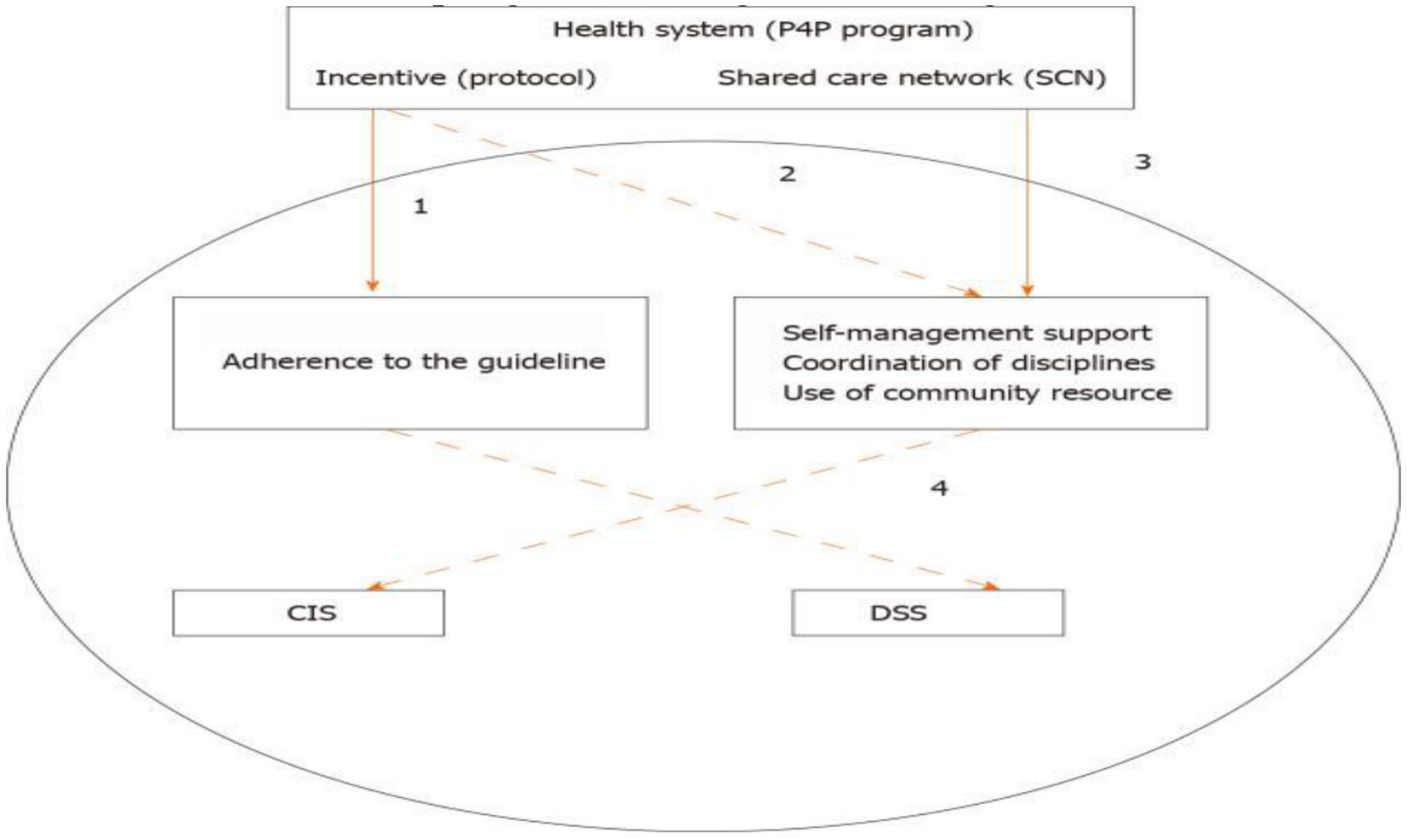
A proposed health system reform centered in adopting care management strategies. (1) Quality management, (2) utilization review & management, (3) health informatic integration and efficiency analysis, (4) engagement & production management, (5) CIS = clinical information system, and (6) DSS = optimizing decision support and administration systems.

Table 1 summarizes the implementation of care management strategies under Taiwan's National Health Insurance (1995–2025). Figure 1 illustrates a proposed reform model centered on integrated care management, supported by clinical information systems (CIS) and decision support systems (DSS).

Together, these approaches advance population health management by aligning risk management, quality management, and patient engagement with informatics-driven efficiency analysis.

## 4 Conclusions

We are pleased to present Volume 2 of this Research Topic, which highlights a diverse range of topics in healthcare research. The editorial team extends its sincere gratitude to the authors and reviewers whose valuable contributions made this volume possible. We anticipate that these articles will encourage global researchers to foster collaborative teams and to explore causal mechanisms that can improve the quality and efficiency of global health strategies. We also hope this editorial will inspire investigators to design and conduct scientific studies using mixed-method approaches. Finally, it is important to emphasize that healthcare services and strategies require not a single solution, but multifaceted and coordinated approaches to achieve sustainable improvements.
